# Association of vitamin D receptor gene polymorphisms and vitamin D levels with asthma and atopy in Cypriot adolescents: a case–control study

**DOI:** 10.1186/s40248-015-0025-0

**Published:** 2015-09-04

**Authors:** Anna Papadopoulou, Panayiotis Kouis, Nikos Middleton, Ourania Kolokotroni, Themistokles Karpathios, Polyxeni Nicolaidou, Panayiotis K. Yiallouros

**Affiliations:** Third Department of Pediatrics, Athens University Medical School, University General Hospital “Attikon”, Athens, Greece; Cyprus International Institute for Environmental & Public Health in association with Harvard School of Public Health, Cyprus University of Technology, Limassol, Cyprus; Department of Nursing, School of Health Sciences, Cyprus University of Technology, Limassol, Cyprus; St George University of London Medical Programme, University of Nicosia, Nicosia, Cyprus; Athens University Medical School, Athens, Greece; Department of Pediatrics, Hospital “Archbishop Makarios III”, Nicosia, Cyprus; 95 Irenes Street, 3041 Limassol, Cyprus

**Keywords:** Vitamin D, VDR, Asthma, Polymorphisms, Adolescents

## Abstract

**Background:**

Polymorphisms in the vitamin D receptor (VDR) gene have been studied in immune-related disorders either as independent contributors or in combination with vitamin D concentration. Vitamin D and VDR have been independently linked to asthma susceptibility. We investigated whether VDR variants were associated independently or in relation to vitamin D levels with asthma in Cypriot adolescents.

**Methods:**

We studied 190 current wheezers, 69 of which were categorized as active asthmatics and 671 healthy controls. We determined three VDR genotypes (*BsmI*, *TaqI*, *ApaI*) and measured serum 25(OH)D levels. Logistic regression and stratified analyses by the presence of hypovitaminosis D (≤20 ng/ml) were used to evaluate the association of the VDR variants with asthma.

**Results:**

The distribution of *TaqI* genotypes was significantly different between controls and current wheezers (*p* = 0.030) or active asthmatics (*p* = 0.014). The tt genotype was over-represented in wheezers (19.2 %) and asthmatics (21.3 %) compared to respective controls (12.9 %). No difference was observed between controls, current wheezers and active asthmatics in the genotypic distribution of *BsmI* and *ApaI* polymorphic sites. After stratification by the presence of hypovitaminosis D, a significant association was detected between tt genotype of *TaqI* polymorphism with wheezing (OR: 1.97, 95 % CI: 1.12, 3.46) and asthma (OR: 2.37, 95CI%: 1.02, 5.52) only in those with normal vitamin D levels (>20 ng/ml) but not in subjects with low vitamin D.

**Conclusions:**

The minor *TaqI* genotype of VDR is associated with asthma in Cypriot adolescents. This polymorphism may contribute to asthma susceptibility primarily under conditions of normal vitamin D levels (>20 ng/ml).

**Electronic supplementary material:**

The online version of this article (doi:10.1186/s40248-015-0025-0) contains supplementary material, which is available to authorized users.

## Background

The role of vitamin D in immune-related disorders has been investigated extensively in the last years. In particular, epidemiological studies have shown a positive link between vitamin D deficiency and asthma susceptibility or asthma characteristics among asthmatic children [1–8]. Nevertheless, several other studies have not replicated theses associations [9–13].

Vitamin D Receptor (VDR) is the mediator of the vitamin D pleiotropic biological actions. The 1,25(OH)2D-VDR complex acts as a nuclear transcription factor which exerts its effects via binding to specific VDR-binding sites of the responding genes, the vitamin D responsive elements [14]. More than 900 genes may be transcribed by VDR [15–17] whereas the ubiquitous expression of the receptor in a wide variety of human tissues potentiates the role of vitamin D in functions beyond the classical skeletal effects [18, 19]. The role of the VDR locus in the development of asthma and allergy is still under investigation.

The activation of the receptor contributes to immune responses via regulation of Th1/Th2 cytokines balance and reduces production of Th2 cytokines (IL-5, IL-10) [20–23]. Furthermore, VDR expression in dendritic cells and activated macrophages has been demonstrated to restrict the inflammatory response and attenuate the severity of allergic phenotypes [24–27]. Additionally to the immuno-modulatory impact, vitamin D has been demonstrated to affect lung structure and function, while its expression has been identified in airway epithelium [28] and bronchial smooth muscle cells [11, 29].

Several studies have examined the association between genetic variants of the VDR and asthmatic populations in different ethnic groups [30–36]. Among the most studied single nucleotide polymorphisms (SNP) are those located in the last intron, (*BsmI* and *ApaI*), and in the last exon (*TaqI*) of the gene. These genetic variations may influence VDR’s RNA stability and translation efficiency and consequently the transcription of the target genes [37–39]. Two family-based association studies conducted in North America populations and two case–control studies, in Chinese Hans and Tunisian populations, showed significant association between one or more of VDR polymorphisms with asthma [30–32, 34]. However, these findings were not replicated in Afro-American [35] or German populations [40]. Most of these studies examined VDR polymorphisms as independent factors for asthma susceptibility. Interestingly, a recent meta-analysis after concluding that *TaqI* and *BsmI* contribute to asthma susceptibility, suggested that this effect could be modified by environmental factors such as levels of serum 25(OH)D [41]. Similar findings have been reported for the associations of VDR polymorphisms with autoimmune disorders [42] and different types of cancer [43–45].

In this study we aimed a) to examine the associations of three well known genetic variants of the VDR gene with wheezing and asthma in a cohort of adolescents in Cyprus and b) to investigate the impact of these polymorphisms in asthma susceptibility in relation to vitamin D status.

## Methods

### Study population

**Fig. 1:**
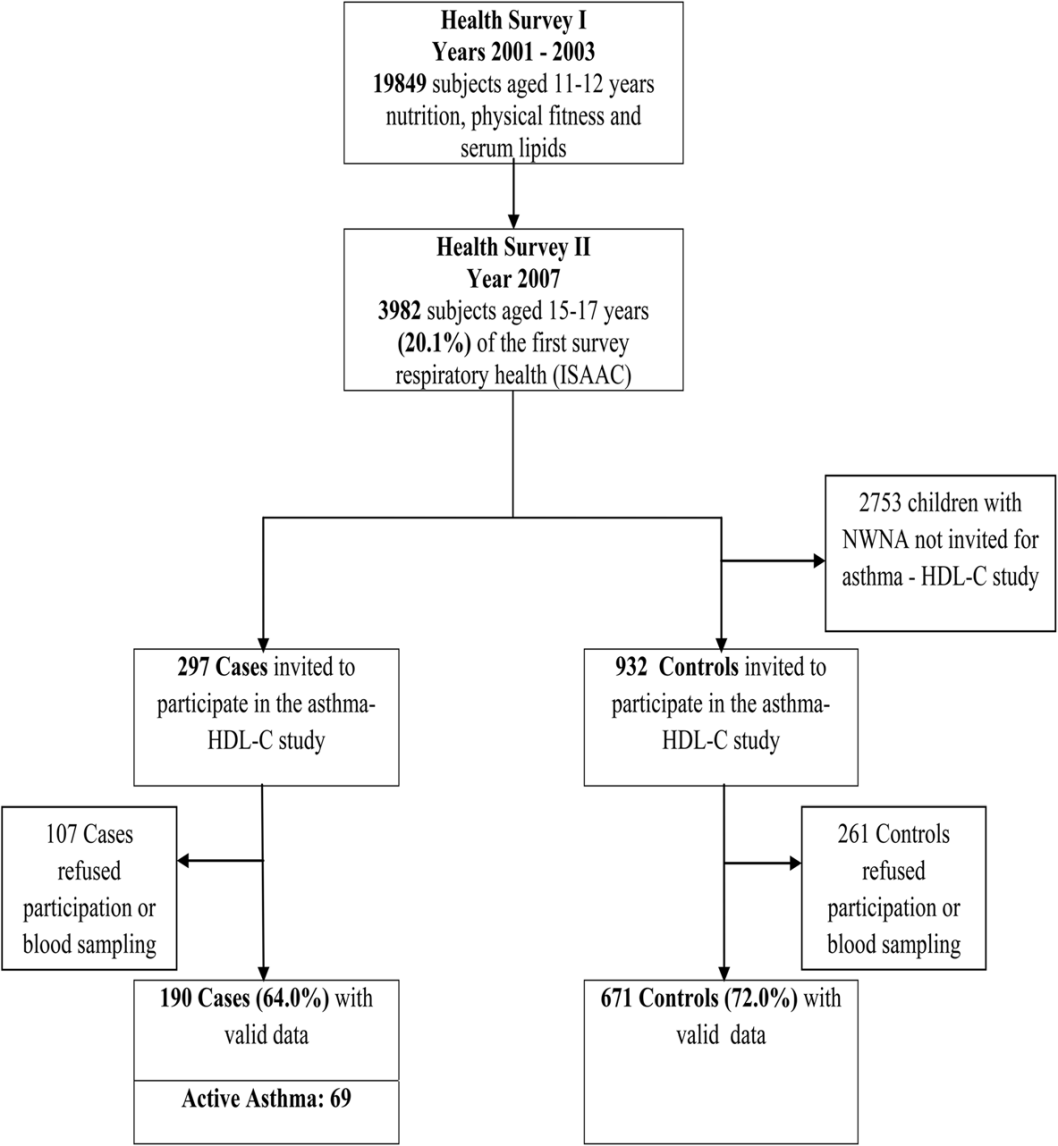
Course of the study: from the 19,849 participants (11–12 years) in Survey 1, a random sample of 3982 adolescents (15–17 years) participated in Survey 2. All (*n* = 297) subjects from the 3982 sample, who reported wheezing in the last 12 months were invited to participate as cases. A sample of 932 children with no diagnosis of asthma and no wheezing in the past 12 months were invited to participate as controls

The participants of this study were selected from a cohort of 3982 children who participated in two large school-based health surveys in Cyprus. The first survey in years 2001–2003 involved all children (*n* = 19,849) attending the 6th form across all primary schools in Cyprus and focused on nutrition and physical fitness. This survey was followed by a second one in year 2007, which recruited 3982 of those participated in the first (20.1 %) and focused on respiratory health (International Study of Asthma and Allergies in Childhood–ISAAC) and risk factors for asthma [46]. In year 2008 when aged 16–18 years and in a case–control design, we invited all those from the 3982 children who on the second survey (ISAAC questionnaire) were current wheezers to participate in this study along with a triplicate number of healthy controls. Current Wheezers were participants who reported wheezing in the past 12 months (Current Wheezers–CUW) and for the purpose of performing a sensitivity analysis, the case definition was further refined to Current Wheezing and Asthma (CUWA), if there was also report of diagnosis of asthma ever. Controls were selected amongst the 3982 adolescents that did not report any wheezing or asthma ever (Never Wheezers Never Asthmatics–NWNA). NWNA were selected using a stratified random sampling approach in order to increase the probability of selection of children at the extremes of BMI change between childhood and adolescence, in line with the scope of another study on the relation of adiposity with asthma. Based on the above selection criteria the group of controls consisted of 671 NWNA and the group of patients of 190 CUW subjects. Among the CUW patients, 69 were categorized as active asthmatic forming the CUWA subgroup (Fig. 1). All participants and their guardians provided informed consent and the study was approved by the National Bioethics Committee of Cyprus.

### Measurements

Collected blood samples were centrifuged on site and serum aliquots were stored at −80 °C until further use. Subsequently, serum levels of 25(OH)D were measured using the enzyme immune assay kit of the Immuno diagnostics Systems Ltd, UK. The intra- and inter-assay coefficients of variation were 12 %.

Atopic sensitization was assessed with skin prick tests (SPT) to 8 common aeroallergens (Greer, USA), (Derp 1 & 2, cat, bahia, mugwort common, grasses, mold mix, olive, weeds and negative (50 % glycerinated saline) and positive (histamine, 1.0 mg/mL) controls) performed and interpreted according to the GA2LEN recommendations [47]. Lung function was assessed through measurement of forced expiratory volume in 1 s (FEV1) and forced vital capacity (FVC) with the help of a spirometer (Vitallograph, UK) and values were expressed as percent of the predicted for the child’s height, age and gender.

### Genotyping

Genomic DNA was isolated from whole blood. We tested three VDR polymorphisms which have been associated with asthma or other pulmonary diseases in several studies among different populations: *TaqI* (rs731236), *BsmI* (rs1544410) and *Apa I* (rs7975232). *TaqI* polymorphism is a synonymous site, a single base change T to C in codon 352 at the 3′ end of the gene. *BsmI* and *ApaI* variants are located in the last intron of the gene resulting from a single base change A to G and G to T respectively. *TaqI* and *ApaI* genotypes were determined on a single 628-bp polymerase chain reaction (PCR) fragment using primers located within intron 8 and exon 9 (5′-CTAGGTCTGGATCCTAAATGCA-3′ and 5′-TTAGGTTGGACAGGAGAGAGAA-3′). *BsmI* genotypes were determined on a 348 bp fragment using the primers 5′-CGGGGAGTATGAAGGACAAA-3′ and 5′-CCATCTCTCAGGCTCCAAAG-3′. Following amplification, the PCR products were subjected to endonuclease digestion for 90 min at 65 °C for *Taq I*, 2 h at 25 °C for *ApaI* and 90 min at 65 °C for *BsmI*. After gel electrophoresis of the restriction products, the different genotypes were distinguished by different fragment sizes. The presence of the restriction site was presented with the lowercase allele (b, t, a) and the absence with the uppercase (B, T, A). Thus, for *TaqI*, the TT genotype resulted in fragments of 433 bp and 195, tt in three fragments of 232, 201 and 195 bp and Tt in four fragments of 433, 232, 201 bp και 195 bp; for *BsmI*, BB resulted in one fragment of 348 bp, bb in two fragments of 242 and 106 bp, and Bb in all three fragments; for *ApaI*, AA resulted in one fragment of 628 bp, aa in two fragments of 477 and 151 bp, and Aa exhibited all three fragments.

Among the analysed 861 samples, *BsmI* polymorphism was not detected in 51 samples (11 CUW, 6 CUWA and 40 controls), *TaqI* polymorphism was not detected in 54 samples (13 CUW, 8 CUWA and 41 controls) and similarly *Apa I* polymorphism was not detected in 52 samples (14 CUW, 8 CUWA and 38 controls) due to technical difficulties. These polymorphisms were considered as missing values and were not included in statistical analysis.

All 3 SNPs were tested for associations with asthmatic status and atopic sensitization.

### Statistical analysis

Participant characteristics of the two cases groups and controls were compared using the chi square test and *t*-test in the case of categorical and continuous variables respectively. Chi-square testing was used for Hardy-Weinberg equilibrium determination. For the investigation of genotypic associations, odd ratios (OR) were reported for the allelic distribution in the study groups. Furthermore, the effect of the homozygous genotypes of the minor alleles was compared against the effect of all other genotypes using binary logistic regression analysis while adjusting for the presence of hypovitaminosis D (≤20 ng/ml). This analysis was also performed separately for participants with and without hypovitaminosis (≤20 ng/ml) in order to assess the potential interaction effect of vitamin D levels on the relationship of genotypes and CUW or CUWA. Stratum specific ORs and the significance level for interaction were reported. All statistical analyses were performed with SPSS statistical package, version 20.0 (IBM, SPSS Inc., Chicago, IL).

## Results

### Study population characteristics

No difference in age and sex distribution was recorded between CUW, CUWA and controls. The mean serum 25(OH)D level in the Control group was 22.9 ng/ml versus 22.94 ng/ml in CUW (*p* > 0.05) and 21.15 ng/ml in CUWA (*p* = 0.017). As expected, SPT positivity at 41.4 and 52 % among CUW and CUWA was significantly higher than the 25 % in Controls (*p* < 0.001; <0.001, respectively) (Table 1). Allergic rhinitis was also much more frequent among CUW (41.5 %) and CUWA (57.4 %) than in Controls (18.4 %) (*p* < 0.001; <0.001, respectively) (Table 1). Main spirometrric indices were not different between study groups.

**Table 1:** Descriptive statistics for the study population

	NWNA (Controls) (*n* = 671)	CUW (Wheezers) (*n* = 190)	*P* value	CUWA (Active Asthmatics) (*n* = 69)	*P* value
Age (yrs)^a^	17.0 (15.9–18.0)	17.0 (16.0–18.1)	0.360	16.9 (15.9–18.1)	0.549
Sex (% male)^b^	40.5 %	43.1 %	0.517	43.5 %	0.626
Vitamin D levels (ng/ml)^a^	22.9 (10.9–35.9)	22.9 (10.5–23.8)	0.946	21.1 (8.8–30.81)	0.017
Sensitization (% positive)^b^	25.7 %	41.4 %	<0.001	52.0 %	<0.001
Rhinitis (% positive)^b^	18.4 %	41.5	<0.001	57.4 %	<0.001
FVC (% predicted)^a^	103.8 (72.7–135.9)	104.9 (77.1–148.3)	0.531	105.6 (78.0–153.6)	0.484
FEV1 (% predicted)^a^	99.3 (69.6–125.3)	99.1 (72.9–130.5)	0.860	97.6 (62.0–125.6)	0.398

*NWNA* Never Wheezing Never Asthma, *CUW* Current Wheezing, *CUWA* Current Wheezing and Asthma

^a^Mean and 95 % CI, Independent sample *t* test for equality of means (2-sited significance)

^b^Percentage, *χ* ^2^ test (asymptomatic 2-sited significance)

### Associations of VDR genotypes with asthma

The genotypes of *TaqI*, *BsmI* and *ApaI* polymorphic sites were in Hardy-Weinberg equilibrium in all study groups. The VDR genetic variants were not associated with 25(OH)D levels in the total population or among controls and patients when examined separately (Additional file 1: Table S1). All SNPs were evaluated for associations with CUW and CUWA status (Table 2). The distribution of the three *TaqI* genotypic groups (TT, Tt, tt) was significantly different between controls and CUW (*p* = 0.030) (Table 2) or CUWA (*p* = 0.014) (Table 3). Particularly, the tt genotype was over-represented in CUW (19.2 %) and CUWA (21.3 %) compared to the respective controls (12.9 %) (OR: 1.59 (95 % CI: 1.02, 2.50); OR: 1.80 (95 % CI: 0.93, 3.48), respectively) No significant difference was observed between controls and CUW or CUWA in the frequencies of the genotypes BB/Bb/bb and AA/Aa/aa of the *BsmI* and *ApaI* polymorphic sites respectively. No association was found between the study groups in the genotypic distribution when we examined the allelic distribution (presence or absence of the minor allele) (Tables 2 & 3).

**Table 2:** Genotypic and allelic association analysis of VDR single-nucleotide polymorphisms between NWNA and CUW

SNP	Genotypic association				Allelic association				
	NWNA (*n*, %)	CUW (*n*, %)	*χ* ^2^	*P* value		NWNA (*n*, %)	CUW (*n*, %)	OR (95 % CI)	*P* value
BsmI									
BB	127 (20.1 %)	38 (21.2 %)							
Bb	327 (51.8 %)	103 (57.5 %)			B	581 (46 %)	179 (50 %)		
bb	177 (28.1 %)	38 (21.2 %)	3.387	0.184	b	681 (54 %)	179 (50 %)	1.17 (0.93–1.48)	0.187
TaqI									
TT	224 (35.6 %)	69 (39 %)							
Tt	325 (51.6 %)	74 (41.8 %)			T	773 (61.3 %)	212 (59.9 %)		
tt	81 (12.9 %)	34 (19.2 %)	7.032	0.030	t	487 (38.7 %)	142 (40.1 %)	0.94 (0.74–1.20)	0.622
ApaI									
AA	232 (36.7 %)	68 (38.6 %)							
Aa	312 (49.3 %)	91 (51.7 %)			A	776 (61.3 %)	227 (64.5 %)		
aa	89 (14.1 %)	17 (9.7 %)	2.343	0.310	a	490 (38.7 %)	125 (35.5 %)	1.15 (0.90–1.47)	0.292

**Table 3:** Genotypic and allelic association analysis of VDR single-nucleotide polymorphisms between NWNA and CUWA

SNP	Genotypic association				Allelic association				
	NWNA (*n*, %)	CUWA (*n*, %)	*χ* ^2^	*P* value		NWNA (*n*, %)	CUWA (*n*, %)	OR (95 % CI)	*P* value
BsmI									
BB	127 (20.1 %)	11 (17.5 %)							
Bb	327 (51.8 %)	32 (50.8 %)			B	581 (46 %)	54 (42.8 %)		
bb	177 (28.1 %)	20 (31.7 %)	0.492	0.782	b	681 (54 %)	72 (57.2 %)	0.88 (0.61–1.27)	0.513
TaqI									
TT	224 (35.6 %)	28 (45.9 %)							
Tt	325 (51.6 %)	20 (32.8 %)			T	773 (61.3 %)	76 (62.3 %)		
tt	81 (12.9 %)	13 (21.3 %)	8.492	0.014	t	487 (38.7 %)	46 (37.7 %)	1.04 (0.71–1.53)	0.922
ApaI									
AA	232 (36.7 %)	19 (31.1 %)							
Aa	312 (49.3 %)	34 (55.7 %)			A	776 (61.3 %)	72 (59 %)		
aa	89 (14.1 %)	8 (13.1 %)	0.966	0.617	a	490 (38.7 %)	50 (41 %)	0.91 (0.62–1.33)	0.628

### TaqI interaction with vitamin D status

The only SNP that was found to be significantly associated with the asthmatic phenotypes, *TaqI* was separately evaluated in participants with and without hypovitaminosis D (serum levels of 25(OH)D ≤20 ng/ml) in order to examine the potential interaction effect of vitamin D status on its association with CUW or CUWA. Although *p* value (0.245) for interaction did not reach statistical significance, we performed a stratified analysis which indicated that the association of TaqI homozygous minor genotype (tt) with CUW was particularly pronounced among the subgroup of participants with normal vitamin D [OR:1.97 (95 % CI:1.12–3.46)]. In contrast, in subjects with low vitamin D the odds ratio estimate [OR:1.13 (95 % CI:0.54–2.38)] was weaker and not statistically significant, indicating a smaller contribution of this subgroup in the observed association (Table 4). A similar result was obtained for the association of genotype tt of *TaqI* with CUWA in the high vitamin D stratum [OR:2.37 (95 % CI: 1.02–5.52)] as opposed to the low vitamin D stratum [OR:1.24 (95 % CI: 0.44–3.54)] (*p*-value for interaction = 0.348) (Table 5).

**Table 4:** Detailed genotypic analysis between NWNA and CUW and between NWNA and CUWA

SNP	NWNA (*n*, %)	CUW (*n*, %)	OR^a^	NWNA (*n*, %)	CUWA (*n*, %)	OR^a^
BsmI						
BB/Bb	454 (71.9 %)	141 (78.8 %)		454 (71.9 %)	43 (68.3 %)	
bb	177 (28.1 %)	38 (21.2 %)	0.70 (0.47–1.04)	177 (28.1 %)	20 (31.7 %)	1.19 (0.68–2.09)
TaqI						
TT/Tt	549 (87.1 %)	143 (80.8 %)		549 (87.1 %)	48 (78.7 %)	
tt	81 (12.9 %)	34 (19.2 %)	1.59 (1.02–2.50)	81 (12.9 %)	13 (21.3 %)	1.80 (0.93–3.48)
ApaI						
AA/Aa	544 (85.9 %)	159 (90.3 %)		544 (85.9 %)	53 (86.9 %)	
aa	89 (14.1 %)	17 (9.7 %)	0.66 (0.38–1.14)	89 (14.1 %)	8 (13.1 %)	0.86 (0.41–1.94)

^a^Adjusted for vitamin deficiency status (<20 ng/ml)

**Table 5:** Stratified genotypic analysis of TaqI among NWNA and CUW and NWNA and CUWA

	Whole population			Low vitamin D stratum (≤20 ng/ml)			High vitamin D stratum (>20 ng/ml)			*P* value for interaction
SNP TaqI	(*n*, %)	(*n*, %)	OR^a^	(*n*, %)	(*n*, %)	OR^a^	(*n*, %)	(*n*, %)	OR^a^	
	NWNA	CUW		NWNA	CUW		NWNA	CUW		
TT/Tt	549 (87.1 %)	143 (80.8 %)		183 (82.4 %)	46 (80.7 %)		357 (89.5 %)	96 (81.4 %)		
tt	81 (12.9 %)	34 (19.2 %)	1.59 (1.02–2.50)	39 (17.6 %)	11 (19.3 %)	1.13 (0.54–2.38)	42 (10.5 %)	22 (18.6 %)	1.97 (1.12–3.46)	0.245
	NWNA	CUWA		NWNA	CUWA		NWNA	CUWA		
TT/Tt	549 (87.1 %)	48 (78.7 %)		183 (82.4 %)	19 (79.2 %)		357 (89.5 %)	29 (78.4 %)		
tt	81 (12.9 %)	13 (21.3 %)	1.80 (0.93–3.48)	39 (17.6 %)	5 (20.8 %)	1.24 (0.44–3.54)	42 (10.5 %)	8 (21.6 %)	2.37 (1.02–5.52)	0.348

^a^Adjusted for vitamin deficiency status (≤20 ng/ml)

## Discussion

In this case–control study of VDR gene variants among Cypriot adolescents, we found that the *TaqI* homozygous minor genotype was associated with wheezing and asthma. Even though there was no significant statistical evidence for effect modification in the *TaqI*-asthma association by vitamin D status, the association appeared stronger among those with higher vitamin D level. No other significant association was detected in the distribution of genotypes and alleles frequencies between asthmatics, wheezers and controls for the *BsmI* and *ApaI* polymorphisms.

Our results on VDR polymorphisms associations with asthma are partly in agreement with the findings of Poon et al. in a family-based cohort of a French-Canadian founder population where *TaqI* and *BsmI* polymorphisms have been associated with asthma in children while *ApaI* has not [32]. Raby et al. [31] tested a total of 7 loci in the VDR gene in individuals from two different cohorts: the Nurses’ Health study population and the Childhood Asthma Management Program (CAMP) population. The association between *TaqI* polymorphism and asthma in the 582 pedigrees selected from the family based Nurses’ Health study population could not be replicated in the case–control study of CAMP. A more recent case–control study performed by Maalmi et al. [34] showed a different distribution of genotype frequencies of VDR variants (i.e. *FokI*, *BsmI* and *TaqI*) between asthmatic and non-asthmatic Tunisian children aged 9 years. On the other hand, three case–control studies conducted in the Chinese Han population investigating the association of genetic variants in the VDR with asthma susceptibility have led to contradictory results [30, 36, 48]. The first was conducted among 1090 individuals including 567 asthmatic patients and among all VDR polymorphisms tested only the *ApaI* marker showed a significant association with asthma [30]. The second study has reported no significant differences in the genotype and allele frequencies of *FokI* and *BsmI* polymorphisms of VDR gene between 101 asthma patients and 206 healthy controls [36]. In the third study all 8 exons of VDR have been sequenced in 467 cases and 288 unrelated healthy controls and failed to find any association between VDR genetic variants and asthma [48].

In a small pilot study conducted among African Americans, Pillai et al. did not find any association of VDR genetic variants with asthma susceptibility [35]; however, within the asthma cohort, six polymorphisms in the VDR gene were significantly associated with quantitative asthma characteristics such as lower baseline spirometric measures and increased IgE levels.

The overall picture of conflicting findings between studies may be due to the phenotypical diversity of asthma and allergy and the different study designs, which assessed different outcomes such as asthma, wheezing, respiratory infections, atopic dermatitis or allergic rhinitis. In addition to the diversity and imprecision in the definition of outcomes, some of the studies lacked statistical power for revealing any associations with specific outcomes. Furthermore, polymorphisms are subject to ethnic variations and geographical differences and the interaction between VDR gene variants with environmental conditions may also differ among populations.

The three tested polymorphisms although are non-functional, they are considered to be linked with other functional polymorphisms and thus participate in a more complex gene network enhancing or inhibiting the expression of VDR target genes [37]. Vitamin D metabolites concentrations may be influenced by VDR polymorphisms as many of the VDR targets are key regulators of vitamin D pathway. Morrison et al. [38] suggested that *BsmI* and *TaqI* VDR polymorphisms define differential transcriptional VDR activity or mRNA stability in vitro. In particular, the BB and tt genotypes have been associated with decreased VDR function and elevated levels of 1,25(OH)2D3.

Although, the optimal level of 25(OH)D [49–51], especially for the non-classical actions of vitamin D, has not yet been defined, low levels of vitamin D are associated with higher risk of having asthma [1–7]. Even though in our population vitamin D levels were significantly lower in asthmatics than in healthy adolescents (*p* < 0.05) there was no association between VDR genetic variants and 25(OH)D levels in the total population or among controls and patients when examined separately. In stratified analyses based on vitamin D deficiency/non-deficiency status, we observed that the tt genotype of the *Taq I* polymorphism was more frequent in wheezers and asthmatics with normal vitamin D levels compared to the respective subgroup of controls. These findings suggest that altered VDR signaling is becoming important mediator of the effects of vitamin D on asthmatic status in the presence of adequate vitamin D levels whereas in the case of vitamin D deficiency the functionality of VDR compared to the overall effect of vitamin D deficiency on asthma risk is not perhaps so significant. Previous studies have reported an interaction between genotype and disease only in conditions of higher or lower exposure to a specific environmental factor, but findings are still inconsistent [52, 53]. Regarding VDR polymorphisms, stronger associations of advanced prostate cancer have been shown with VDR genotypes ff and AA of the polymorphisms *FokI* and *CDX*-*2* respectively, in the presence of adequate levels of ultraviolet radiation [54]. In contrast to our findings, TaqI tt genotype was found to have no or a protective effect for prostate cancer under adequate levels of sun exposure [54, 55].

There are a number of limitations. The use of self-reported questionnaire data to define the study outcomes is not as accurate as the use of clinical parameters. However, most of the previously published studies have also used epidemiological definitions for asthma. In this study we initially used the looser case definition of Current Wheezers (CUW–report of wheezing in the past 12 months) and then we performed a sensitivity analysis, where the case definition was further refined with the combination of Current Wheezing and report of Asthma (CUWA). The estimates we found for TaqI with the stricter asthma definition were in the same direction and magnitude with those found with the looser definition although there was not always statistical significance due to the smaller sample size. Although misclassification in defining the disease status is more likely in epidemiological definitions of asthma, we do not think that this could have been influenced by the type of VDR genetic variants of the subjects and thus confound the recovered associations. If anything, the noise in the data accompanying the epidemiological definition of asthma would have pushed the significance of our estimates towards the null. Although we adjusted the associations of VDR genotypes with asthma for vitamin D status, we cannot exclude the presence of residual confounding by variables not measured with our questionnaires.

## Conclusions

In conclusion, we found an association of the minor *TaqI* genotype with asthma in Cypriot teenagers. We also observed that the minor *TaqI* genotype is probably not an independent factor for asthma susceptibility but it assumes its mediating role in the association of the disease under certain environmental conditions such as normal vitamin D concentrations. Further studies are needed to confirm this finding in larger populations and reveal the functional mechanisms implicated in the interaction of vitamin D with asthma and VDR genotype expression.

## Additional file

Additional file 1: Table S1. Comparison of vitamin D levels distribution among VDR genetic variants in controls and patients. (DOCX 16 kb)

## Abbreviations

CUW: Current Wheezers
CUWA: Current Wheezing and Asthma
ISAAC: International Study of Asthma and Allergies in Childhood
NWNA: Never Wheezers Never Asthmatics
SPT: Skin prick tests
VDR: Vitamin D receptor

## References

[1] Bener A, Ehlayel MS, Tulic MK, Hamid Q (2012). Vitamin D deficiency as a strong predictor of asthma in children. Int Arch Allergy Immunol.

[2] Checkley W, Robinson CL, Baumann LM, Hansel NN, Romero KM, Pollard SL (2015). 25-hydroxy vitamin D levels are associated with childhood asthma in a population-based study in Peru. Clin Exp Allergy.

[3] Niruban SJ, Alagiakrishnan K, Beach J, Senthilselvan A (2014). Association of vitamin D with respiratory outcomes in Canadian children. Eur J Clin Nutr.

[4] van Oeffelen AA, Bekkers MB, Smit HA, Kerkhof M, Koppelman GH, Haveman-Nies A (2011). Serum micronutrient concentrations and childhood asthma: the PIAMA birth cohort study. Pediatr Allergy Immunol.

[5] Hollams EM, Hart PH, Holt BJ, Serralha M, Parsons F, de Klerk NH (2011). Vitamin D and atopy and asthma phenotypes in children: a longitudinal cohort study. Eur Respir J.

[6] Brehm JM, Acosta-Perez E, Klei L, Roeder K, Barmada M, Boutaoui N (2012). Vitamin D insufficiency and severe asthma exacerbations in Puerto Rican children. Am J Respir Crit Care Med.

[7] Uysalol M, Mutlu LC, Saracoglu GV, Karasu E, Guzel S, Kayaoglu S (2013). Childhood asthma and vitamin d deficiency in Turkey: is there cause and effect relationship between them?. Ital J Pediatr.

[8] Kolokotroni O, Papadopoulou A, Middleton N, Kouta C, Raftopoulos V, Nicolaidou P (2015). Vitamin D levels and status amongst asthmatic and non-asthmatic adolescents in Cyprus: a comparative cross-sectional study. BMC Public Health.

[9] Tolppanen AM, Sayers A, Granell R, Fraser WD, Henderson J, Lawlor DA (2013). Prospective association of 25-hydroxyvitamin D3 and D2 with childhood lung function, asthma, wheezing, and flexural dermatitis. Epidemiology.

[10] Yao TC, Tu YL, Chang SW, Tsai HJ, Gu PW, Ning HC (2014). Suboptimal vitamin D status in a population-based study of Asian children: prevalence and relation to allergic diseases and atopy. PLoS One.

[11] Bosse Y, Maghni K, Hudson TJ (2007). 1alpha,25-dihydroxy-vitamin D3 stimulation of bronchial smooth muscle cells induces autocrine, contractility, and remodeling processes. Physiol Genomics.

[12] Krobtrakulchai W, Praikanahok J, Visitsunthorn N, Vichyanond P, Manonukul K, Pratumvinit B (2013). The effect of vitamin D status on pediatric asthma at a university hospital, Thailand. Allergy Asthma Immunol Res.

[13] Tolppanen AM, Williams D, Henderson J, Lawlor DA (2011). Serum 25-hydroxy-vitamin D and ionised calcium in relation to lung function and allergen skin tests. Eur J Clin Nutr.

[14] Haussler MR, Jurutka PW, Hsieh JC, Thompson PD, Selznick SH, Haussler CA (1995). New understanding of the molecular mechanism of receptor-mediated genomic actions of the vitamin D hormone. Bone.

[15] Wang TT, Tavera-Mendoza LE, Laperriere D, Libby E, MacLeod NB, Nagai Y (2005). Large-scale in silico and microarray-based identification of direct 1,25-dihydroxyvitamin D3 target genes. Mol Endocrinol.

[16] Walters MR (1992). Newly identified actions of the vitamin D endocrine system. Endocr Rev.

[17] Holick MF (2008). The vitamin D deficiency pandemic and consequences for nonskeletal health: mechanisms of action. Mol Aspects Med.

[18] Rosen CJ, Adams JS, Bikle DD, Black DM, Demay MB, Manson JE (2012). The nonskeletal effects of vitamin D: an endocrine society scientific statement. Endocr Rev.

[19] Pludowski P, Holick MF, Pilz S, Wagner CL, Hollis BW, Grant WB (2013). Vitamin D effects on musculoskeletal health, immunity, autoimmunity, cardiovascular disease, cancer, fertility, pregnancy, dementia and mortality–a review of recent evidence. Autoimmun Rev.

[20] Cantorna MT, Woodward WD, Hayes CE, DeLuca HF (1998). 1,25-dihydroxyvitamin D3 is a positive regulator for the two anti-encephalitogenic cytokines tgf-beta 1 and IL-4. J Immunol.

[21] van Etten E, Mathieu C (2005). Immunoregulation by 1,25-dihydroxyvitamin D3: basic concepts. J Steroid Biochem Mol Biol.

[22] Boonstra A, Barrat FJ, Crain C, Heath VL, Savelkoul HF, O’Garra A (2001). 1alpha,25-dihydroxyvitamin D3 has a direct effect on naive CD4(+) t cells to enhance the development of Th2 cells. J Immunol.

[23] Vasiliou JE, Lui S, Walker SA, Chohan V, Xystrakis E, Bush A (2014). Vitamin D deficiency induces Th2 skewing and eosinophilia in neonatal allergic airways disease. Allergy.

[24] Adams JS, Hewison M (2008). Unexpected actions of vitamin D: new perspectives on the regulation of innate and adaptive immunity. Nat Clin Pract Endocrinol Metab.

[25] Adams JS, Sharma OP, Gacad MA, Singer FR (1983). Metabolism of 25-hydroxyvitamin D3 by cultured pulmonary alveolar macrophages in sarcoidosis. J Clin Invest.

[26] Adorini L, Penna G, Giarratana N, Roncari A, Amuchastegui S, Daniel KC (2004). Dendritic cells as key targets for immunomodulation by vitamin D receptor ligands. J Steroid Biochem Mol Biol.

[27] Provvedini DM, Tsoukas CD, Deftos LJ, Manolagas SC (1983). 1,25-dihydroxyvitamin D3 receptors in human leukocytes. Science.

[28] Hansdottir S, Monick MM, Hinde SL, Lovan N, Look DC, Hunninghake GW (2008). Respiratory epithelial cells convert inactive vitamin D to its active form: potential effects on host defense. J Immunol.

[29] Damera G, Fogle HW, Lim P, Goncharova EA, Zhao H, Banerjee A (2009). Vitamin D inhibits growth of human airway smooth muscle cells through growth factor-induced phosphorylation of retinoblastoma protein and checkpoint kinase 1. Br J Pharmacol.

[30] Saadi A, Gao G, Li H, Wei C, Gong Y, Liu Q (2009). Association study between vitamin D receptor gene polymorphisms and asthma in the Chinese Han population: a case-control study. BMC Med Genet.

[31] Raby BA, Lazarus R, Silverman EK, Lake S, Lange C, Wjst M (2004). Association of vitamin D receptor gene polymorphisms with childhood and adult asthma. Am J Respir Crit Care Med.

[32] Poon AH, Laprise C, Lemire M, Montpetit A, Sinnett D, Schurr E (2004). Association of vitamin D receptor genetic variants with susceptibility to asthma and atopy. Am J Respir Crit Care Med.

[33] Ismail MF, Elnady HG, Fouda EM (2013). Genetic variants in vitamin D pathway in Egyptian asthmatic children: a pilot study. Hum Immunol.

[34] Maalmi H, Sassi FH, Berraies A, Ammar J, Hamzaoui K, Hamzaoui A (2013). Association of vitamin D receptor gene polymorphisms with susceptibility to asthma in Tunisian children: a case control study. Hum Immunol.

[35] Pillai DK, Iqbal SF, Benton AS, Lerner J, Wiles A, Foerster M (2011). Associations between genetic variants in vitamin D metabolism and asthma characteristics in young African Americans: a pilot study. J Investig Med.

[36] Fang WL, Gao LB, Liang WB, Xue H, Bai P, Lv ML (2009). Association analysis of vitamin D receptor gene polymorphisms in Chinese population with asthma. Iran J Allergy Asthma Immunol.

[37] Uitterlinden AG, Fang Y, Van Meurs JB, Pols HA, Van Leeuwen JP (2004). Genetics and biology of vitamin D receptor polymorphisms. Gene.

[38] Morrison NA, Qi JC, Tokita A, Kelly PJ, Crofts L, Nguyen TV (1994). Prediction of bone density from vitamin D receptor alleles. Nature.

[39] Whitfield GK, Remus LS, Jurutka PW, Zitzer H, Oza AK, Dang HT (2001). Functionally relevant polymorphisms in the human nuclear vitamin D receptor gene. Mol Cell Endocrinol.

[40] Vollmert C, Illig T, Altmuller J, Klugbauer S, Loesgen S, Dumitrescu L (2004). Single nucleotide polymorphism screening and association analysis–exclusion of integrin beta 7 and vitamin D receptor (chromosome 12q) as candidate genes for asthma. Clin Exp Allergy.

[41] Tizaoui K, Berraies A, Hamdi B, Kaabachi W, Hamzaoui K, Hamzaoui A (2014). Association of vitamin D receptor gene polymorphisms with asthma risk: systematic review and updated meta-analysis of case-control studies. Lung.

[42] Li K, Shi Q, Yang L, Li X, Liu L, Wang L (2012). The association of vitamin D receptor gene polymorphisms and serum 25-hydroxyvitamin D levels with generalized vitiligo. Br J Dermatol.

[43] Li H, Stampfer MJ, Hollis JB, Mucci LA, Gaziano JM, Hunter D (2007). A prospective study of plasma vitamin D metabolites, vitamin D receptor polymorphisms, and prostate cancer. PLoS Med.

[44] Mikhak B, Hunter DJ, Spiegelman D, Platz EA, Hollis BW, Giovannucci E (2007). Vitamin d receptor (vdr) gene polymorphisms and haplotypes, interactions with plasma 25-hydroxyvitamin D and 1,25-dihydroxyvitamin D, and prostate cancer risk. Prostate.

[45] Kim HS, Newcomb PA, Ulrich CM, Keener CL, Bigler J, Farin FM (2001). Vitamin D receptor polymorphism and the risk of colorectal adenomas: evidence of interaction with dietary vitamin D and calcium. Cancer Epidemiol Biomarkers Prev.

[46] Yiallouros PK, Savva SC, Kolokotroni O, Behbod B, Zeniou M, Economou M (2012). Low serum high-density lipoprotein cholesterol in childhood is associated with adolescent asthma. Clin Exp Allergy.

[47] Bousquet J, Heinzerling L, Bachert C, Papadopoulos NG, Bousquet PJ, Burney PG (2012). Practical guide to skin prick tests in allergy to aeroallergens. Allergy.

[48] Li F, Jiang L, Willis-Owen SA, Zhang Y, Gao J (2011). Vitamin D binding protein variants associate with asthma susceptibility in the Chinese Han population. BMC Med Genet.

[49] Ross AC, Manson JE, Abrams SA, Aloia JF, Brannon PM, Clinton SK (2011). The 2011 report on dietary reference intakes for calcium and vitamin D from the institute of medicine: what clinicians need to know. J Clin Endocrinol Metab.

[50] Holick MF, Binkley NC, Bischoff-Ferrari HA, Gordon CM, Hanley DA, Heaney RP (2011). Evaluation, treatment, and prevention of vitamin D deficiency: an endocrine society clinical practice guideline. J Clin Endocrinol Metab.

[51] Heaney RP (2013). Health is better at serum 25(OH)D above 30 ng/ml. J Steroid Biochem Mol Biol.

[52] Simpson A, John SL, Jury F, Niven R, Woodcock A, Ollier WE (2006). Endotoxin exposure, CD14, and allergic disease: an interaction between genes and the environment. Am J Respir Crit Care Med.

[53] Colilla S, Nicolae D, Pluzhnikov A, Blumenthal MN, Beaty TH, Bleecker ER (2003). Evidence for gene-environment interactions in a linkage study of asthma and smoking exposure. J Allergy Clin Immunol.

[54] Bodiwala D, Luscombe CJ, French ME, Liu S, Saxby MF, Jones PW (2004). Polymorphisms in the vitamin d receptor gene, ultraviolet radiation, and susceptibility to prostate cancer. Environ Mol Mutagen.

[55] John EM, Schwartz GG, Koo J, Van Den Berg D, Ingles SA (2005). Sun exposure, vitamin D receptor gene polymorphisms, and risk of advanced prostate cancer. Cancer Res.

