# Supplementary material for: Association of vitamin D receptor gene polymorphisms and vitamin D levels with asthma and atopy in Cypriot adolescents: a case–control study
Source: Multidiscip Respir Med. 2015 Sep 4;10(1):26. doi: 10.1186/s40248-015-0025-0 (PMC4559891; doi:10.1186/s40248-015-0025-0)
Supplement: Additional file 1: Table S1. — Comparison of vitamin D levels distribution among VDR genetic variants in controls and patients. (DOCX 16 kb) [file 40248_2015_25_MOESM1_ESM.docx]

Table S1: Comparison of vitamin D levels distribution among VDR genetic variants in controls and patients.

|  | **NWNA**  **(Controls)** | | | **CUW**  **(Wheezers)** | | | **CUWA**  **(Active Asthmatics)** | | |
| --- | --- | --- | --- | --- | --- | --- | --- | --- | --- |
|  | **Vitamin D levels†**  **(ng/ml)** | **F**  **statistic‡** | **P Value‡** | **Vitamin D levels†**  **(ng/ml)** | **F**  **statistic‡** | **P Value‡** | **Vitamin D levels†**  **(ng/ml)** | **F**  **statistic‡** | **P Value‡** |
|  |  |  |  |  |  |  |  |  |  |
| BsmI |  |  |  |  |  |  |  |  |  |
| BB | 22.4 (21.3 - 23.5) |  |  | 22.5 (20.5 - 24.6) |  |  | 21.4 (17.6 - 25.3) |  |  |
| Bb | 23.1 (22.4 - 23.8) |  |  | 22.9 (21.7 - 24.2) |  |  | 20.3 (18.5 - 22.1) |  |  |
| Bb | 22.5 (21.6 - 23.5) | 0.758 | 0.469 | 24.1 (22.0 - 26.1) | 0.624 | 0.537 | 23.3 (20.2 - 26.1) | 1.668 | 0.197 |
|  |  |  |  |  |  |  |  |  |  |
| TaqI |  |  |  |  |  |  |  |  |  |
| TT | 22.8 (22.0 - 23.6) |  |  | 23.6 (22.1 - 22.0) |  |  | 21.4 (19.5 - 23.3) |  |  |
| Tt | 23.1 (22.4 - 23.8) |  |  | 22.8 (21.2 - 24.3) |  |  | 20.5 (17.3 - 23.6) |  |  |
| Tt | 21.8 (20.5 - 23.1) | 1.351 | 0.260 | 22.7 (20.7 - 24.6) | 0.353 | 0.703 | 22.0 (18.7 - 25.3) | 0.320 | 0.727 |
|  |  |  |  |  |  |  |  |  |  |
| ApaI |  |  |  |  |  |  |  |  |  |
| AA | 22.7 (21.9 - 23.6) |  |  | 23.4 (22.0 - 24.9) |  |  | 21.8 (19.6 - 24.0) |  |  |
| Aa | 23.2 (22.5 - 23.9) |  |  | 23.1 (21.8 - 24.4) |  |  | 21.2 (19.2 - 23.2) |  |  |
| aa | 22.0 (20.6 - 23.4) | 1.166 | 0.312 | 21.1 (17.3 - 24.9) | 0.986 | 0.375 | 19.8 (13.5 - 26.1) | 0.341 | 0.712 |
|  |  |  |  |  |  |  |  |  |  |
| †Mean and 95% CI,  ‡One-Way Anova test for equality of means | | | |  |  |  |  |  |  |
|  | | | |  |  |  |  |  |  |
